# Design, Synthesis and Evaluation of Naphthalimide Derivatives as Potential Anticancer Agents for Hepatocellular Carcinoma

**DOI:** 10.3390/molecules22020342

**Published:** 2017-02-22

**Authors:** Chaochao Ge, Liping Chang, Ying Zhao, Congcong Chang, Xiaojuan Xu, Haoying He, Yuxia Wang, Fujun Dai, Songqiang Xie, Chaojie Wang

**Affiliations:** 1 Pharmaceutical College, Henan University, Kaifeng 475001, China; gechao1234365@163.com (C.G.); 15890173281@163.com (Y.Z.); xuxiaojuahn@163.com (X.X.); hhy0926@foxmail.com (H.H.); 2 Key Laboratory of Natural Medicine and Immuno-Engineering, Henan University, Kaifeng 475001, China; changlip@126.com (L.C.); kuffychang@163.com (C.C.); wcjsxq@henu.edu.cn (C.W.); 3 College of Chemistry and Chemical Engineering, Henan University, Kaifeng 475001, China; 4 Institute of Chemical Biology, Henan University, Kaifeng 475001, China

**Keywords:** synthesis, naphthalimide, hepatocellular carcinoma, cell cycle, lung metastasis

## Abstract

Two kinds of naphthalimide derivatives were synthesized and evaluated for in vitro their anti-hepatocellular carcinoma properties. Compound **3a** with a fused thiazole fragment to naphthalimide skeleton inhibited cell migration of SMMC-7721 and HepG2, and further in vivo trials with two animal models confirmed that compound **3a** moderately inhibited primary H22 tumor growth (52.6%) and potently interrupted lung metastasis (75.7%) without obvious systemic toxicity at the therapeutic dose. Mechanistic research revealed that compound **3a** inhibited cancerous liver cell growth mostly by inducing G2/M phase arrest. Western blotting experiments corroborated that **3a** could up-regulate the cell cycle related protein expression of cyclin B1, CDK1 and p21, and inhibit cell migration by elevating the E-cadherin and attenuating integrin α6 expression. Our study showed that compound **3a** is a valuable lead compound worthy of further investigation.

## 1. Introduction

Hepatocellular carcinoma (HCC), the most common malignancy of the liver, is the third most common cause of cancer-related deaths in the world [1,2]. At present, some feasible and curative measures, including resection and liver transplant, are used in treating HCC. However, once patients are diagnosed with HCC, the disease is often already at an advanced stage, and accompanied with micrometastases. In this case, surgical therapy is no longer a curative treatment option. Hence, traditional chemotherapy treatment is irreplaceable and can be used alone or in combination with other therapies. Postoperative chemotherapy might improve survival time by reducing tumor size and eradicating micrometastases [3], however, current high HCC-associated mortality indicates that the design and synthesis of highly efficient antitumor agents which exert greater efficacy to HCC without obvious toxicity remain of significant importance [4].

**Figure 1:**
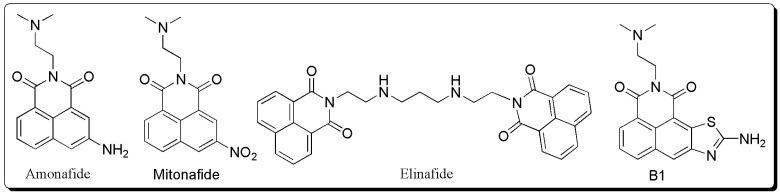
The structures of amonafide, mitonafide, elinafide and **B1**.

In the field of antitumor agents, naphthalimide derivatives remain one of the most important classes of drug candidates. Naphthalimide analogs have been considered as a promising group of anticancer agents. Amonafide, mitonafide and elinafide (Figure 1) have reached the clinical trials stage for the treatment of solid tumors and exhibited excellent anti-tumor activity [5,6,7], but most of them were abandoned because of various adverse effects such as dose-limiting bone marrow toxicity [8,9]. Accordingly, extensive efforts including the modification of the side chain, aromatic ring system, and the substituents on the ring have been attempted to search for more selective naphthalimide derivatives to improve the potency and reduce the adverse effects [10,11]. Braña et al. and Qian et al. have designed and synthesized several series of heterocyclic fused naphthalimide derivatives. They showed that some compounds exhibited better activity than amonafide [12,13,14,15]. In the excellent paper, Qian and co-workers reported a new series of naphthalimide derivatives containing the 2-aminothiazole moiety. Among these derivatives compound **B1** (Figure 1) was found to induce expression of tumor suppressor gene p53 in HeLa cells and MCF-7 cell lines, increase the activity of p53 and induce apoptosis in a caspase-independent manner. However, there are no studies on this kind of compounds in vivo [16,17].

Our group has also made significant attempts in synthesizing naphthalimide-polyamine derivatives to enhance cytotoxicity [18,19]. In order to compare the biological activities of heterocyclic fused naphthalimide derivatives and derivatives with straight chain substituents on the naphthalene ring system, naphthalimide derivatives with formyl alkyl esters as substituents on naphthalimide skeleton and aminothiazole fused naphthalimide-polyamine conjugates were synthesized in this paper for establishing better structure activity relationship (SAR). These novel-synthesized compounds were evaluated for their in vitro and in vivo activities in comparison with amonafide.

## 2. Results and Discussion

### 2.1. Synthesis

**Scheme 1:**
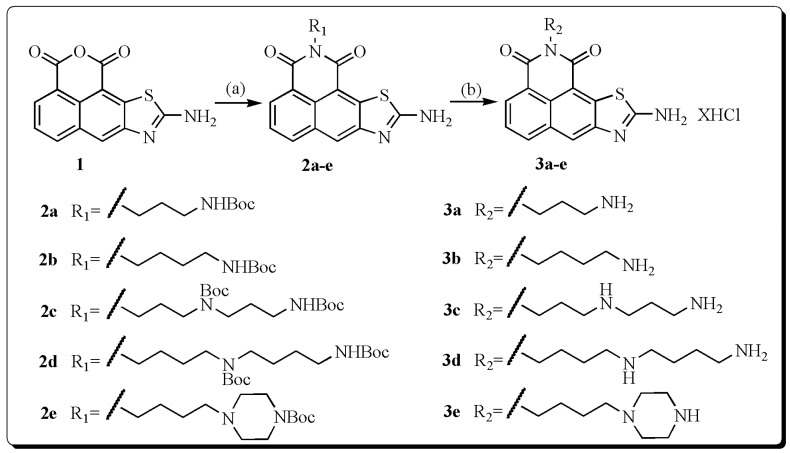
Synthetic protocol for target compounds **3a**–**e**.* Reagents and Conditions*: (**a**) R_1_NH_2_, EtOH reflux, 2.5 h; (**b**) EtOH, 4 M HCl, room temperature, overnight.

The general route for the synthesis of compounds **3a**–**e** with a thiazo moiety fused to a naphthalimide skeleton is illustrated in Scheme 1.

Compound **1** was prepared by a previously reported procedure [14]. Without further purification the crude compound **1** was condensed with corresponding amines R_1_NH_2_ (the Boc protected polyamines were prepared by a modified procedure reported previously [20]) to give the mixture containing compounds **2a**–**e**. After purification by flash column chromatography, pure intermediates **2a**–**e** were mixed with 4 M HCl at room temperature to obtain the target compounds **3a**–**e** as hydrochloride salts.

**Scheme 2:**
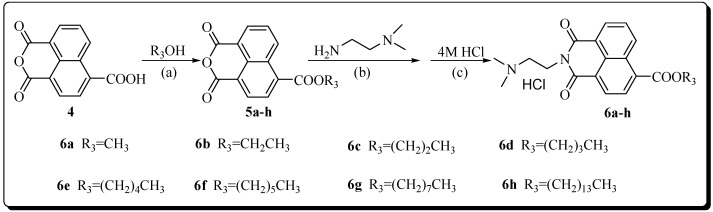
Synthetic protocol of target compounds **6a**–**h**. *Reagents and Conditions*: (**a**) R_3_OH, H_2_SO_4_, reflux, 4 h; (**b**) (CH_3_)_2_NCH_2_CH_2_NH_2_, EtOH, reflux 1 h; (**c**) EtOH, 4M HCl, room temperature, overnight.

The synthesis of target compounds **6a**–**h** with formyl alkyl esters at the 4-position of naphthalimide was performed as shown in Scheme 2. Intermediate **4** was prepared by a modified previously reported procedure [21,22]. 4-Carboxy-1,8-naphthalic anhydride (**4**) was esterified with the corresponding alcohol in the presence of H_2_SO_4_ to afford products **5a**–**h**. Products **5a**–**h** were condensed with 2-dimethylethylaminoethylamine to give crude imides, which were purified by flash column chromatography. These intermediates were finally mixed with 4 M HCl at room temperature to obtain the target compounds **6a**–**h** as hydrochloride salts.

### 2.2. Biological Evaluation

#### 2.2.1. Antitumor Activity In Vitro

The in vitro anticancer activities of target compounds were evaluated against four human tumor cell lines (SMMC-7721: human hepatoma cell line, HepG2: human hepatoma cell line, HCT-116: human colorectal cancer cell line and K562: human leukemia cell line) by using standard 3-(4,5-dimethylthiazol-2-yl)-2,5-diphenyltetrazolium bromide (MTT) assays after treatment for 48 h. Amonafide was employed as the reference drug. The antiproliferative results of the preliminary evaluation were shown in Table 1.

The results showed that polyamine conjugates **3a**–**e** with a 2-aminothiazole group fused to the naphthalimide skeleton were active for all tested tumor cells. Compound **3c** displayed best inhibition potency against two hepatoma cells, and compound **3a** also exhibited a little bit improved anti-tumor activity compared to amonafide. Among compounds **6a**–**h** with formyl alkyl esters as substituents, compounds **6a** and **6b** showed the best antiproliferative activity than other analogues without activity (IC_50_ > 50 µM), indicating that their anticancer activity was sensitive to the length of the alkyl chain. Therefore, compounds **3a** and **3c** were selected for further anti-tumor investigation.

#### 2.2.2. Anti-Tumor Activity In Vivo

**Figure 2:**
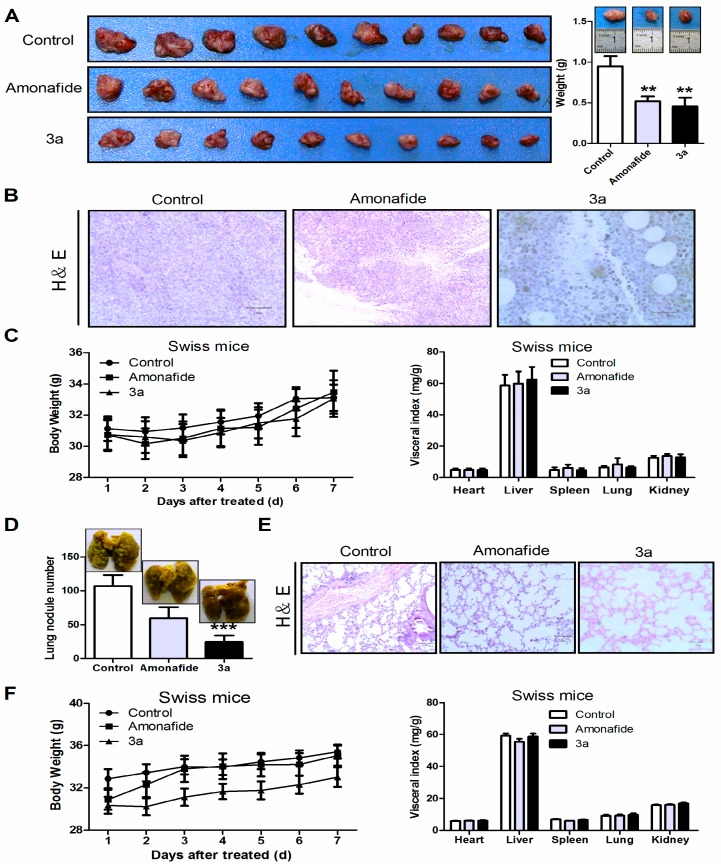
Antitumor activity of compound **3a** was evaluated in vivo. (**A**) Photographs of tumor obtained from each treatment group excised on day 10. (*n* = 3, x ± SD, ** *p* < 0.01) (left); Mean tumor weight with representative photo correspondingly (right); (**B**) Representative photograph of histological section was obtained from each treatment group excised on day 10 (HE stain, 20×). The scale bar represents 100 µm; (**C**) The changes of body weight of mice treated with **3a**, amonafide, and normal saline. Visceral indexes (heart, liver, spleen, lung and kidney) were evaluated after treatment in with **3a**, amonafide, and normal saline; (**D**) Lung metastasis nodules numbers for pulmonary metastasis in mice treatment with **3a**, amonafide, and normal saline. (*n* = 3, x ± SD, *** *p* < 0.001); (**E**) Representative photograph of histological section was obtained from each treatment group excised on day 10 (H&E staining, 20×); (**F**) The changes of body weight of mice treated with **3a**, amonafide, and normal saline. Visceral indexes (heart, liver, spleen, lung and kidney) were evaluated after treatment in with **3a**, amonafide, and normal saline.

To further evaluate the antitumor activity of new compounds in vivo, we chose two H22 (mice hepatoma cell line) tumor transplant models: solid tumor (tumor growth inhibition evaluation) and pulmonary metastasis tumor (tumor metastasis evaluation). Compounds **3a** and **3c** were selected for pre-trials to determine the maximum tolerated dose (MTD), and animals had a better tolerance for **3a**, while **3c** displayed some acute toxicity. Therefore, in vivo trials of **3a** and amonafide as a reference drug were conducted. As shown in Figure 2A, the tumor volumes in the treated group were smaller than those of the negative control group. Similarly, the mean weight of tumors in the compound **3a**-treated group was reduced by 52.63% compared with the control group (0.95 ± 0.12 g vs. 0.45 ± 0.11 g; *n* = 10), while the tumor inhibitory rate of amonafide was 45.26% (0.52 ± 0.06 g, *n* = 10) (Figure 2A). Consistent with this, the histological examination revealed that obvious morphological changes and necrosis of tumor cells were observed in compound **3a** and amonafide groups (Figure 2B). During the experiment, the average weight of mice increased slightly. Compared with the control group, no significant difference in visceral indexes (heart, liver, spleen, lung and kidney) was observed in compound **3a** (Figure 2C).

Compared with the mice treated with normal saline, mice treated with **3a** displayed few metastases and the inhibitory rate was 75.73% (Figure 2D). Amonafide, as the reference drug, moderately decreased lung metastasis nodules numbers (40.7%). Consistent with these results, the alveolar structure of mice in compound **3a** group tended to be normal while the negative control group alveolar spaces were filled with cancer cells as shown in the histological section (Figure 2E). For systemic toxicity evaluation, as shown in Figure 2F, compound **3a** had no obvious adverse effect on body weight and visceral indexes of heart, liver, spleen, lung as well as kidney.

Therefore, compound **3a** could not only inhibit the primary tumor growth, but also prevent the pulmonary metastasis of H22 cells in Swiss mice more potently than amonafide. In another aspect, compound **3a** at the therapeutic dose displayed favorable systemic toxicity in the preliminary toxicology evaluation, which was equally a critical factor for further development.

#### 2.2.3. **3a**-Induced Cell Morphology Changes and Apoptosis

**Figure 3:**
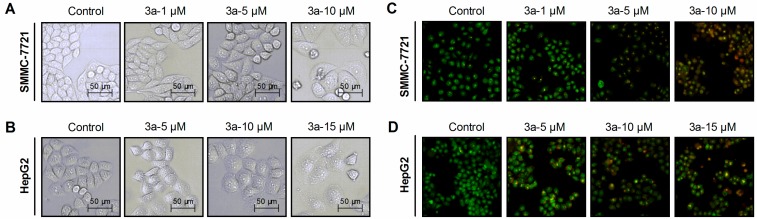
The morphology of SMMC-7721 (**A**); and HepG2 cells (**B**) treated with compound **3a** of various concentrations for 48 h. Cells were photographed under an inverted biological microscope (20×); Cell membrane integrity and nuclear structure of SMMC-7721 cells (**C**); and HepG2 cells (**D**) treated with compound **3a** of various concentrations for 24 h by AO/EB staining using HCS (20×). The experiments were repeated three times and representative images are shown.

To investigate the inhibitory effect of compound **3a**, we first observed the cell size and shape in SMMC-7721 and HepG2 cells. Cell morphology changes indicate that many physiological processes are affected, such as cell cycle, adhesion and migration [23,24]. Compound **3a** caused significant shape changes including cell rounding and cell volume increasing, and these alterations were induced by compound **3a** in a dose-dependent manner (Figure 3A,B).

Apoptosis is characterized by specific morphological and biochemical features including chromatin condensation, cell shrinkage, activation of caspase and loss of mitochondrial membrane potential [25,26,27]. It has been reported that naphthalimide derivatives exerted antitumor activity via different death mechanisms. Xie, S.Q. et al. [28] reported that a novel amonafide analogue NPC-16 not only induced HepG2 cell apoptosis but also autophagy. Furthermore, some novel naphthalimide derivatives induced tumor cell apoptosis via lysosomal membrane permeabilization [29]. Based on these studies, AO/EB staining experiment by high content screening (HCS) [30] was conducted to determine whether compound **3a** could induce SMMC-7721 and HepG2 cells apoptosis. In the negative control group, green fluorescent appeared to be uniform and both SMMC-7721 and HepG2 cells showed normal structures (Figure 3C,D). After treated with compound **3a**, SMMC-7721 cells and HepG2 cells showed membrane blebbing and apoptotic-like nuclei fragmentation. Meanwhile, the orange fluorescence (AO/EB) was enhanced at the high dose. These results showed that compound **3a** at high dose induced apoptosis.

#### 2.2.4. **3a**-Induced G2/M Phase Arrest

**Figure 4:**
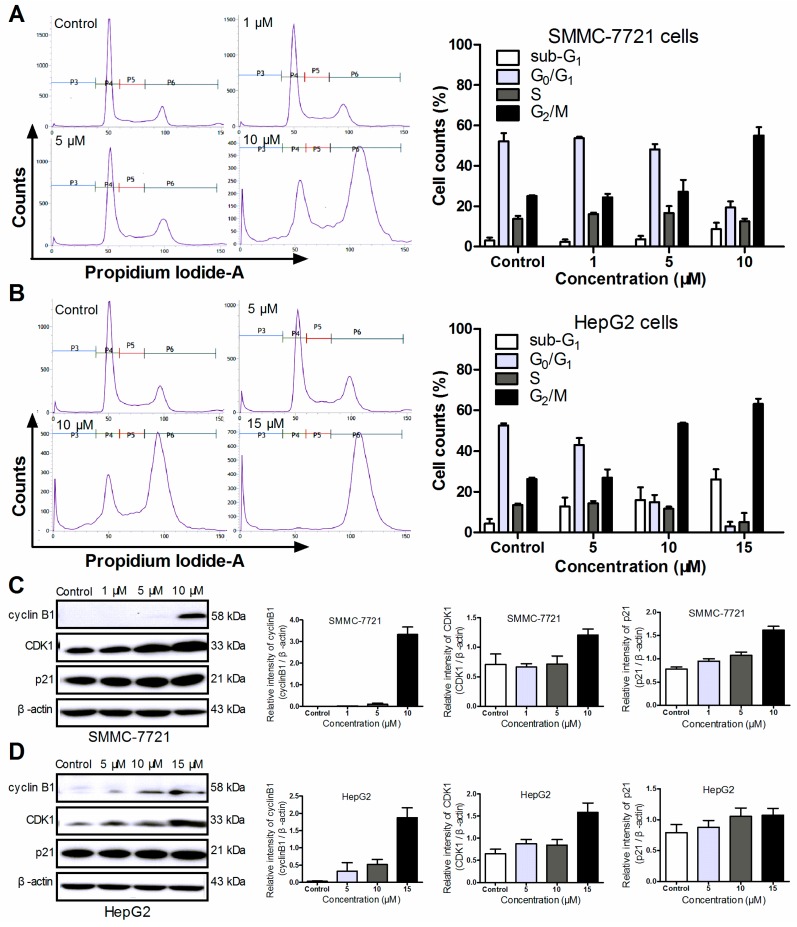
Cell cycle distribution was analyzed after compound **3a** treatment for 48 h. (**A**) The effect of compound **3a** on cell cycle was detected in SMMC-7721 cells. Cell cycle was significantly arrested in G2/M phase in a dose-dependent manner P3: sub-G1, P4: G_0_/G_1_, P5: S, P6: G_2_/M; (**B**) The effect of compound **3a** on cell cycle was detected in HepG2 cells. Cell cycle was significantly arrested in G2/M phase in a dose-dependent manner; (**C**) The expression levels of cell cycle-related proteins, including cyclin B1, CDK1 and p21, were analyzed by western blotting in SMMC-7721 cells; (**D**) The expression levels of cell cycle-related proteins, including cyclin B1, CDK1 and p21, were analyzed by western blotting in HepG2 cells. All of the experiments were repeated three times for each group and the other results were consistent.

The cell cycle plays an important role in the cell, leading to its division. With the progress of the cell cycle, cells shape changes from flat to spherical and increasing volume is filled with DNA, RNA, enzymes and proteins [23]. Cells’ morphological changes tend to signify cell cycle events. Many studies showed that cell cycle arrest at different cell cycle points was accompanied by distinct morphological changes [23]. Previous researches showed that many drugs induced cell rounding and G2/M cell cycle arrest in cancer cells [31,32]. To determine whether cell cycle arrest occurred, cell cycle phase distribution was detected by flow cytometry. We found that G2/M phase cell population increased significantly with increasing concentrations of compound **3a** in both SMMC-7721 (Figure 4A) and HepG2 (Figure 4B) cells. In addition, the sub-G1 hypodiploid cell population increased with increasing concentrations of compound **3a**, representing the apoptosis induced by **3a**. However, G2/M phase cell cycle arrest seems to play a more important role than apoptosis in **3a**-induced tumor cell inhibition.

To further uncover the potential molecular mechanism of **3a**-induced G2/M arrest, the expression levels of cell cycle-related proteins, including cyclin B1, CDK1 and p21 were analyzed by western blotting. The results revealed that the expression of cyclin B1 and CDK1 was strongly up-regulated compared with the control groups (Figure 4C,D). Meanwhile, the expression of p21 was up-regulated after treatment with compound **3a** (Figure 4C,D).

It has been reported that the amount of cyclin B1 and the activity of the cyclin B1-CDK1 complex which was named maturation promoting factor or mitosis promoting factor (MPF) increased through the cell cycle until mitosis, where they fell abruptly due to degradation of cyclin B1 [33,34]. In this study, we found that expression of cyclin B1 and CDK1 were both up-regulated in a dose-dependent manner (Figure 4C,D), which was similar to the effects of 6-methoxy-3-(3′,4′,5′-trimethoxybenzoyl)-1*H*-indole (BPR0L075) on colorectal cancer cells [35]. These results suggested that compound **3a** might induce cell cycle arrest by up-regulated cyclin B1 and CDK1 expression. The cyclin kinase inhibitor p21, negative growth regulator of the cell cycle, played an important role in inducing G1 or G2/M cell cycle phase arrest [36,37]. We speculated that p21 involved in the effect of compound **3a** on cell cycle arrest. In this study, the expression of p21 was up-regulated in hepatoma cells after treatment of compound **3a** (Figure 4C,D). We suspected that the p21 bound to and inhibited the activity of CDK1 or cyclinB1-CDK1 complexes, which resulted in the induction of cell cycle arrest.

#### 2.2.5. **3a**-Induced Inhibition of Migration and Invasion

**Figure 5:**
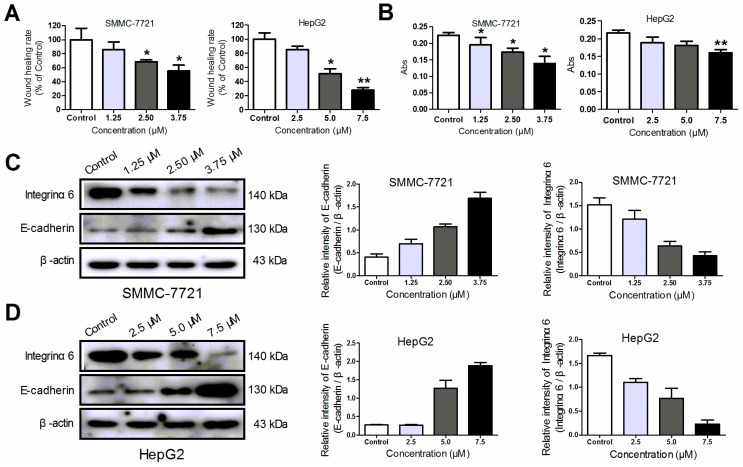
The compound **3a** inhibited invasion and migration of SMMC-7721 and HepG2 cells. (**A**) **3a** at different concentrations inhibited SMMC-7721 (1.25, 2.5, 3.75 µM) and HepG2 (2.5, 5.0, 7.5 µM) cells migration (*n* = 3, x ± SD, * *p* < 0.05, ** *p* < 0.01); (**B**) Transwell inserts coated with matrigel were used to examine the invasion ability of SMMC-7721 and HepG2 cells treated with compound **3a**. Representative images were shown and cell invasion ability was expressed by the optical density value (*n* = 3, x ± SD, * *p* < 0.05, ** *p* < 0.01); (**C**) The expression levels of E-cadherin and integrin α6 in SMMC-7721 cells were measured using western blotting; (**D**) The expression levels of E-cadherin and integrin α6 in HepG2 cells were measured using western blotting. All of the experiments were repeated three times for each group and the other results were consistent.

Compared with the control group, a scratch assay showed that the migratory ability of cells was inhibited after SMMC-7721 and HepG2 cells were treated with compound **3a** (Figure 5A). A transwell invasion assay indicated that fewer invaded cells were observed after the treatment of compound **3a** at high concentration, compared with the negative control group (Figure 5B). Taken together, our results indicated that compound **3a** was able to decrease the migration ability of SMMC-7721 and HepG2 cells in vitro. To further uncover the potential mechanism underlying tumor metastasis, the migration-related protein E-cadherin and integrin α6 were tested by western blotting. The expression of E-cadherin was up-regulated while integrin α6 was down-regulated with increasing concentration of compound **3a** in SMMC-7721 and HepG2 cells (Figure 5C,D). These results testified that compound **3a** inhibited cancer cells migration by up-regulating E-cadherin expression and down-regulating integrin α6 expression.

Cell migration and invasion are the most prominent features of malignant cell behavior. Many reports [38,39] showed that naphthalimide derivatives could inhibit tumor cells migration and invasion in vivo. In this study, compound **3a** inhibited migration and invasion of SMMC-7721 and HepG2 cells using scratch assay and transwell invasion assay in vitro. E-cadherin, a tumor-suppressor gene, was reported for being related to cell–cell adhesion and tumor-cell invasion and metastasis [40]. It was reported that integrin α6, a member of integrin family, was up-regulated obviously and positively correlated with invasion ability in malignant tumors including HCC [41]. In this study, the expression of E-cadherin and integrin α6 were altered with the increasing concentration of compound **3a** (Figure 5C,D), suggesting that E-cadherin and integrin α6 played a key role in the mechanism through which compound **3a** inhibited the migration and invasion ability of SMMC-7721 and HepG2 cells.

## 3. Materials and Methods

### 3.1. General Information

All solvents and reagents were acquired from suppliers and used without further purification. All ^1^H-NMR and ^13^C-NMR spectra were recorded on an AV-400 model spectrometer (Bruker BioSpin, Zürich, Switzerland) in D_2_O, CDCl_3_, DMSO-*d*_6_ or CD_3_OD and chemical shifts for ^1^H-NMR spectra were reported in parts per million with reference to residual solvent protons. High resolution mass spectrometry was performed on a Q-TOF with ESI ionisation. ESI-MS spectrum (low resolution) was recorded on an ESQUIRE-LC mass spectrometer (Agilent, Palo Alto, CA, USA). The target compounds with the purity being higher than 95% were analyzed using combustion analysis, performed on a GmbH Vario EL elemental instrument (Elementar, Langenselbold, Germany), and results were within 0.4% of theoretical values.

### 3.2. General Procedure for the Synthesis of Compounds ***3a**–**e***

Intermediate **1** was prepared by a procedure reported previously [14]. To a suspension of compound **1** (2 mmol) in ethanol (30 mL) was added the corresponding amine R_1_NH_2_ (2 mmol). The reaction mixture was refluxed for 3 h and monitored by TLC. After completion of the reaction, the ethanol was removed by a rotary evaporator and then the residue was purified by careful column chromatography to obtain Boc-protected intermediates **2a**–**e**.

*2-Amino-[N-(3-butoxycarbonylaminopropyl)]-benz[de]thiazo[4,5-g]isoquinoline-1,3(2H)-dione* (**2a**). Yield: 54%, ^1^H-NMR (DMSO-*d*_6_) δ: 8.32–8.37 (m, 2H, Ar-H), 8.24–8.26 (m, 1H, Ar-H), 8.01 (s, 2H, C-NH, CO-NH), 7.61 (t, *J* = 8.00 Hz, 1H, Ar-H), 4.04 (t, *J* = 8.00 Hz, 2H), 2.98–3.03 (m, 2H), 1.72–1.79 (m, 2H), 1.37 (s, 9H, 3 × CH_3_); ESI-MS *m*/*z*: 427.14 [M + 1]^+^; MW: 426.49.

*2-Amino-[N-(4-(butoxycarbonylpiperazin-1-yl)butyl]-benz[de]thiazo[4,5-g]isoquinoline-1,3(2H)-dione* (**2e**). Yield: 52%, ^1^H-NMR (DMSO-*d*_6_) δ: 8.35 (t, *J* = 8.26 Hz, 2H, Ar-H), 8.26 (d, *J* = 8.28 Hz, 1H, Ar-H), 8.02 (s, 2H, C-NH, CO-NH), 7.82 (t, *J* = 7.82 Hz, 1H, Ar-H), 4.04 (t, *J* = 7.22 Hz, 2H, 1 × CH_2_), 3.25–3.26 (m, 4H, 2 × CH_2_), 2.25–2.32 (m, 6H, 3 × CH_2_), 1.60–1.67 (m, 2H, 1 × CH_2_), 1.44–1.52 (m, 2H, 1 × CH_2_), 1.37 (s, 9H, 3 × CH_3_); ESI-MS *m*/*z*: 510.21 [M + 1]^+^; MW: 509.62.

The respective *N*-Boc protected intermediates **2a**–**e** (1.0 mmol) were dissolved in EtOH (10 mL) and stirred at 0 °C for 10 min. Then 4 M HCl was added dropwise at 0 °C. The reaction mixture was stirred at room temperature overnight. The solutions typically gave a white solid precipitate. The solid was filtered, washed several times with absolute ethanol, and dried under vacuum to give the pure target compounds **3a**–**e**.

*2-Amino-[N-(3-aminopropyl)]-benz[de]thiazo[4,5-g]isoquinoline-1,3(2H)-dione dihydrochloride* (**3a**). Yield: 77%, ^1^H-NMR (D_2_O) δ: 7.68 (d, *J* = 9.04 Hz, 1H), 7.12–7.16 (m, 2H), 6.98 (d, *J* = 3.92 Hz, 1H), 3.71 (t, *J* = 7.22 Hz, 2H), 2.98 (t, *J* = 9.62 Hz, 2H), 1.86 (t, *J* = 10.10 Hz, 2H); ^13^C-NMR (D_2_O) δ: 168.73, 163.90, 163.47, 144.47, 131.12, 129.61, 129.10, 127.42, 123.91, 121.56, 120.31, 119.08, 118.00, 37.47, 37.19, 25.29; HRMS (TOF MS ESI, [M − 2HCl + H]^+^): *m*/*z* calculated for C_16_H_14_N_4_O_2_S: 326.0837, found: 327.0912. Anal. Calcd. for C_16_H_16_Cl_2_N_4_O_2_S·2.2H_2_O: C 43.78%, H 4.68%, N 12.76%; found C 43.53%, H 4.68%, N 12.48%; MW: 435.76.

*2-Amino-[N-(4-aminobutyl)]-benz[de]thiazo[4,5-g]isoquinoline-1,3(2H)-dione dihydro-chloride* (**3b**). Yield: 74%, ^1^H-NMR (D_2_O) δ: 7.40 (d, *J* = 9.00 Hz, 1H, Ar-H), 6.94 (t, *J* = 9.78 Hz, 1H, Ar-H), 6.83 (d, *J* = 9.84 Hz, 1H, Ar-H), 6.60 (d, *J* = 4.4 Hz, 1H, Ar-H),3.38 (t, 2H, *J* = 9.26 Hz, 1 × CH_2_), 2.94 (t, *J* = 10.54 Hz, 2H, 1 × CH_2_), 1.55–1.63 (m, 2H, 1 × CH_2_), 1.37 (t, *J* = 1.18 Hz, 2H, 1 × CH_2_); ^13^C-NMR (D_2_O) δ: 168.27, 162.83, 162.28, 140.76, 129.26, 128.85, 128.55, 127.75, 123.16, 121.03, 119.97, 118.22, 117.13, 39.41, 38.88, 24.34, 23.98; HRMS (TOF MS ESI, [M − 2HCl + H]^+^): *m*/*z* calculated for C_17_H_16_N_4_O_2_S: 340.0994, found: 341.1078. Anal. Calcd. for C_17_H_18_Cl_2_N_4_O_2_S·3.5H_2_O: C 42.86%, H 5.29%, N 11.76%; found C 42.73%, H 5.05%, N 11.58%; MW: 449.78.

*2-Amino-{N-[3-(3-aminopropylamino)-propyl]}-benz[de]thiazo[4,5-g]isoquinoline-1,3(2H)-dione trihydrochloride* (**3c**). Yield: 78%, ^1^H-NMR (D_2_O) δ: 7.65 (d, *J* = 9.20 Hz, 1H, Ar-H), 7.06–7.17 (m, 2H, Ar-H), 6.89 (d, *J* = 3.20 Hz, 1H, Ar-H), 3.65 (t, *J* = 9.80 Hz, 2H, 1 × CH_2_), 3.01–3.13 (m, 6H, 3 × CH_2_), 1.99–2.09 (m, 2H, 1 × CH_2_), 1.84 (t, *J* = 10.01 Hz, 2H, 1 × CH_2_); ^13^C-NMR (D_2_O) δ: 168.48, 163.38, 162.84, 141.16, 129.59, 129.31, 129.12, 127.94, 123.61, 121.60, 120.32, 118.53, 117.67, 45.43, 44.73, 37.58, 36.57, 24.14, 23.71; HRMS (TOF MS ESI, [M − 3HCl + H]^+^): *m*/*z* calculated for C_19_H_21_N_5_O_2_S: 383.1416, found: 384.1497. Anal. Calcd. for C_19_H_24_Cl_3_N_5_O_2_S·3H_2_O: C 41.73%, H 5.53%, N 12.81%; found C 41.91%, H 5.28%, N 12.79%; MW: 529.31.

*2-Amino-{N-[4-(4-aminobutylamino)-butyl]}-benz[de]thiazo[4,5-g]isoquinoline-1,3(2H)-dione trihydrochloride* (**3d**). Yield: 76%, ^1^H-NMR (D_2_O) δ: 7.58–7.66 (m, 1H, Ar-H), 7.08–7.18 (m, 2H, Ar-H), 7.01 (s, 1H, Ar-H), 3.60 (t, *J* = 8.46 Hz, 2H, 1 × CH_2_), 2.97–3.05 (m, 6H, 3 × CH_2_), 1.68–1.70 (m, 6H, 3 × CH_2_), 1.52 (t, *J* = 5.66 Hz, 2H,); ^13^C-NMR (D_2_O) δ: 168.69, 163.65, 163.24, 143.18, 130.22, 129.39, 129.21, 127.62, 123.85, 121.63, 120.46, 118.58, 118.41, 47.12, 46.90, 39.87, 38.79, 24.15, 23.94, 23.24, 22.81; HRMS (TOF MS ESI, [M − 3HCl + H]^+^): *m*/*z* calculated for C_21_H_25_N_5_O_2_S: 411.1729, found: 412.1800; Anal. Calcd. for C_21_H_28_Cl_3_N_5_O_2_S·H_2_O: C 46.80%, H 5.61%, N 13.00%; found C 46.71%, H 5.48%, N 12.96%; MW: 557.36.

*2-Amino-[N-(4-(piperazin-1-yl)butyl]-benz[de]thiazo[4,5-g]isoquinoline-1,3(2H)-dione trihydrochloride* (**3e**). Yield: 75%, ^1^H-NMR (D_2_O) δ: 7.48 (d, *J* = 9.12 Hz, 1H, Ar-H), 6.93–7.05 (m, 2H, Ar-H), 6.71 (d, *J* = 3.44 Hz, 1H, Ar-H), 3.23–3.82 (m, 12H), 1.67–1.74 (m, 2H), 1.40 (t, *J* = 11.42 Hz, 2H); ^13^C-NMR (D_2_O) δ: 168.33, 162.91, 162.36, 140.11, 129.50, 128.96, 128.28, 127.98, 123.36, 121.30, 120.16, 118.56, 117.07, 56.53, 48.40, 40.71, 39.72, 23.88, 20.93; HRMS (TOF MS ESI, [M − 3HCl + H]^+^): *m*/*z* calculated for C_21_H_23_N_5_O_2_S: 409.1572, found: 410.1645; Anal. Calcd. for C_21_H_26_Cl_3_N_5_O_2_S·0.4H_2_O: C 47.94%, H 5.13%, N 13.31%; found C 47.93%, H 5.25%, N 13.29%; MW: 555.35.

### 3.3. General Procedure for the Synthesis of Compounds ***6a**–**h***

To a suspension of 4-carboxyl-1,8-naphthalic anhydride (**4**) [21,22] (4 mmol) in the corresponding alcohol (8 mmol), sulfuric acid (0.4 mL, 96%) was added at room temperature. Then the mixture was allowed to stir under reflux for 4 h. After completion of the reaction, the slurry was poured to crushed ice. The product **5a**–**h** was obtained by filtration and washed with water. The wet product was dried on vacuum and used for the next step without further purification.

*4-Carboxylic acid n-butyl ester-1H-benz[de]isoquinoline-1,3(2H)-dione* (**5d**). Yield: 87%, ^1^H-NMR (DMSO-*d*_6_) δ: 9.11 (dd, *J*_1_ = *J*_2_ = 1.06 Hz, 1H), 8.58–8.61 (m, 2H), 8.38 (d, *J* = 7.6 Hz, 1H), 8.01–8.05 (m, 1H), 4.45 (t, *J* = 6.56 Hz, 2H), 1.76–1.80 (m, 2H), 3.08 (s, 6H), 1.44–1.50 (m, 2H), 0.96 (t, *J* = 7.4 Hz, 3H); ESI-MS *m*/*z*: 299.09 [M + 1]^+^; MW: 298.29.

To a suspension of **5a**–**h** (2.0 mmol) in ethanol (20 mL) *N*,*N*-dimethylethylenediamine (2 mmol). was added. This mixture was refluxed for 2 h. After completion, the solvent was removed in a rotary evaporator to obtain the residue. After purified by careful column chromatography the residue (1.0 mmol) was dissolved in EtOH (10 mL) and stirred at 0 °C for 10 min. Then 4 M HCl was added dropwise at 0 °C. The reaction mixture was stirred at room temperature for 1 h. The solution typically gave a white solid as a precipitate. The solid was filtered, washed several times with absolute ethanol, and dried under vacuum to give the pure target compounds **6a**–**h**.

*2-[(2-Dimethylamino)ethyl]-4-carboxylic acid methyl ester-1H-benz[de]isoquinoline-1,3(2H)-dione hydrochloride* (**6a**). Yield: 55%, ^1^H-NMR (CD_3_OD) δ: 9.21 (d, *J* = 11.6 Hz, 1H), 8.63 (d, *J* = 10.06 Hz, 2H), 8.40 (d, *J* = 3.04 Hz, 1H), 7.94 (t, *J* = 10.84 Hz, 1H), 4.58 (t, *J* = 15.64 Hz, 2H), 4.09 (s, 3H), 3.68 (t, *J* = 8.06 Hz, 2H), 3.08 (s, 6H); ^13^C-NMR (D_2_O) δ: 167.33, 164.39, 163.92, 132.60, 132.25, 131.69, 129.97, 129.91, 128.32, 127.96, 126.64, 123.20, 120.22, 54.99, 53.31, 43.32, 35.30; ESI-MS *m*/*z*: 327.12 [M + 1 − HCl]^+^. Anal. Calcd. for C_18_H_19_ClN_2_O_4_·0.6H_2_O: C 57.86%, H 5.45%, N 7.50%; found C 57.77%, H 5.74%, N 7.49%; MW: 362.81.

*2-[(2-Dimethylamino)ethyl]-4-carboxylic acid ethyl ester-1H-benz[de]isoquinoline-1,3(2H)-dione hydrochloride* (**6b**). Yield: 59%. ^1^H-NMR (D_2_O) δ: 8.03 (d, *J* = 11.64 Hz, 1H), 7.78 (d, *J* = 9.72 Hz, 1H), 7.55 (d, *J* = 10.40 Hz, 1H), 7.39 (d, *J* = 10.44 Hz, 1H,), 7.17 (t, *J* = 10.86 Hz, 1H), 4.12–4.26 (m, 4H), 3.40 (t, *J* = 9.04 Hz, 2H), 2.94 (s, 6H), 1.30 (t, *J* = 9.56 Hz, 3H), ^13^C-NMR (D_2_O) δ: 166.32, 163.91, 163.32, 132.22, 131.93, 131.41, 129.61, 129.54, 128.12, 127.39, 126.12, 122.69, 119.83, 63.09, 54.72, 43.30, 35.19, 13.38; ESI-MS *m*/*z*: 341.11 [M + 1 − HCl]^+^; Anal. Calcd. for C_19_H_21_ClN_2_O_4_·1.1H_2_O: C 57.53%, H 5.90%, N 7.06%; found C 57.47%, H 5.92%, N 7.01%; MW: 376.83.

*2-[(2-Dimethylamino)ethyl]-4-carboxylic acid n-propyl ester-1H-benz[de]isoquinoline-1,3(2H)-dione hydro-chloride* (**6c**). Yield: 42%, ^1^H-NMR (CD_3_OD) δ: 8.95 (d, *J* = 11.6 Hz, 1H), 8.40 (d, *J* = 9.68 Hz, 2H), 8.32 (d, *J* = 3.04 Hz, 1H), 7.72 (t, *J* = 10.68 Hz, 1H); 4.51 (t, *J* = 8.06 Hz, 2H), 4.42 (t, *J* = 8.88 Hz, 2H), 3.60 (t, *J* = 8.02 Hz, 2H), 3.08 (s, 6H), 1.86–1.93 (m, 2H),1.11 (t, *J* = 9.92 Hz, 3H); ^13^C-NMR (CD_3_OD) δ: 165.30, 163.99, 163.54, 132.69, 132.12, 131.04, 129.54, 129.07, 127.94, 127.89, 124.80, 121.99, 67.32, 55.65, 42.83, 35.29, 21.75, 9.62; ESI-MS *m*/*z*: 355.16 [M + 1 − HCl]^+^; Anal. Calcd. for C_20_H_23_ClN_2_O_4_: C 61.46%, H 5.93%, N 7.17%; found C 61.24%, H 5.97%, N 7.13%; MW: 390.86.

*2-[(2-Dimethylamino)ethyl]-4-carboxylic acid n-butyl ester-1H-benz[de]isoquinoline-1,3(2H)-dione hydrochloride* (**6d**). yield: 57%, ^1^H-NMR (400 MHz, CD_3_OD) δ: 8.99 (d, *J* = 11.52 Hz, 1H), 8.43 (d, *J* = 9.56 Hz, 1H), 8.37 (d, *J* = 3.44 Hz, 1H), 8.16 (d, *J* = 10.12 Hz, 1H), 7.76 (t, *J* = 10.64 Hz, 1H), 4.54–4.45 (m, 4H), 3.60 (t, *J* = 7.90 Hz, 2H), 3.08 (s, 6H), 1.92–1.83 (m, 2H), 1.62–1.52 (m, 2H), 1.08 (t, *J* = 12.00 Hz, 3H); ^13^C-NMR (CD_3_OD, 400 MHz) δ: 166.82, 164.04, 163.89, 132.75, 132.16, 131.07, 129.56, 129.14, 127.97, 124.97, 121.90, 65.56, 55.71, 42.83, 35.29, 30.46, 19.04, 12.79; ESI-MS *m*/*z*: 369.18 [M + 1 − HCl]^+^; Anal. Calcd. for C_21_H_25_ClN_2_O_4_·0.24H_2_O: C 61.64%, H 6.28%, N 6.85%; found C 61.88%, H 6.58%, N 6.87%; MW: 404.89.

*2-[(2-dimethylamino)ethyl]-4-carboxylic acid n-amyl ester-1H-benz[de]isoquinoline-1,3(2H)-dione hydrochloride* (**6e**). Yield: 54%,^1^H-NMR (CD_3_OD) δ: 9.20 (d, *J* = 11.4 Hz, 1H), 8.63–8.61 (m, 2H), 8.37 (d, *J* = 10.04 Hz, 1H), 7.91 (d, *J* = 10.68 Hz, 1H), 4.54 (t, *J* = 7.76 Hz, 2H), 4.47 (t, *J* = 9.06 Hz, 2H), 3.56 (t, *J* = 7.76 Hz, 2H), 3.04 (s, 6H), 1.86 (t, *J* = 9.32 Hz, 2H), 1.48–1.40 (m, 4H), 0.96 (t, *J* = 9.16 Hz, 3H); ^13^C-NMR (CD_3_OD) δ: 165.81, 164.03, 163.60, 132.75, 131.06, 129.55, 129.14, 127.94, 124.97, 121.95, 65.83, 55.70, 42.82, 35.30, 29.10, 29.05, 22.09, 13.05; ESI-MS *m*/*z*: 383.17 [M + 1 − HCl]^+^; Anal. Calcd. for C_22_H_27_ClN_2_O_4_·0.25H_2_O: C 62.41%, H 6.55%, N 6.62%; found C 62.67%, H 6.72%, N 6.62%; MW: 418.91.

*2-[(2-Dimethylamino)ethyl]-4-carboxylic acid n-hexyl ester-1H-benz[de]isoquinoline-1,3(2H)-dione hydrochloride* (**6f**). Yield: 43%, ^1^H-NMR (-CD_3_OD) δ: 9.06 (d, *J* = 11.16 Hz, 1H), 8.52–8.45 (m, 2H), 8.24 (d, *J* = 9.84 Hz, 1H), 7.81 (d, *J* = 10.48 Hz, 1H), 4.56–4.45 (m, 4H), 3.59 (t, *J* = 7.28 Hz, 2H), 3.08 (s, 6H), 1.89 (t, *J* = 9.78 Hz, 2H), 1.57–1.41 (m, 6H), 0.97 (t, *J* = 5.56 Hz, 3H); ^13^C-NMR (CD_3_OD-) δ: 165.90, 164.13, 163.70, 132.92, 132.24, 131.13, 129.63, 129.56, 129.26, 129.11, 127.97, 124.99, 122.09, 65.96, 55.94, 42.96, 35.31, 31.29, 29.35, 25.53, 22.28, 13.03; ESI-MS *m*/*z*: 397.21 [M + 1 − HCl]^+^; Anal. Calcd. for C_23_H_29_ClN_2_O_4_·0.3H_2_O: C 63.02%, H 6.81%, N 6.39%; found C 62.96%, H 6.69%, N 6.47%; MW: 432.94.

*2-[(2-Dimethylamino)ethyl]-4-carboxylic acid n-octyl ester-1H-benz[de]isoquinoline-1,3(2H)-dione hydrochloride* (**6g**). Yield: 47%, ^1^H-NMR (CD_3_OD) δ: 9.15 (t, J = 13.56 Hz, 1H), 8.62–8.53 (m, 2H), 8.35–8.31 (m, 1H), 7.90 (t, J = 10.82 Hz, 1H), 4.57 (t, J = 7.64 Hz, 2H), 4.50 (t, J = 8.88 Hz, 2H), 3.60 (t, J = 7.84 Hz, 2H), 3.08 (s, 6H), 1.94–1.85 (m, 2H), 1.57–1.35 (m, 10H), 0.93 (t, J = 8.56 Hz, 3H); ^13^C-NMR (CD_3_OD) δ: 165.80, 164.04, 163.60, 132.17, 132.19, 131.08, 129.56, 129.17, 127.99, 127.94, 124.99, 121.98, 65.84, 55.72, 42.83, 35.30, 31.63, 29.04, 28.40, 25.89, 22.37, 13.13. ESI-MS m/z: 425.24 [M + 1 − HCl]^+^; Anal. Calcd. for C_25_H_33_ClN_2_O_4_·0.2H_2_O: C 64.63%, H 7.25%, N 6.03%; found C 64.89%, H 7.23%, N 6.06%; MW: 460.99.

*2-[(2-Dimethylamino)ethyl]-4-carboxylic acid n-tetradecyl ester-1H-benz[de]isoquinoline -1,3(2H)-dione hydrochloride* (**6h**). Yield: 47%, ^1^H-NMR (CD_3_OD) δ: 9.18 (d, *J* = 11.56 Hz, 1H), 8.60 (t, *J* = 10.46 Hz, 2H), 8.35 (d, *J* = 10.32 Hz, 1H), 7.90 (t, *J* = 10.76 Hz, 1H), 4.57 (t, *J* = 7.82 Hz, 2H), 4.49 (t, *J* = 8.89 Hz, 2H), 3.59 (t, *J* = 7.90 Hz, 2H), 3.08 (s, 6H), 1.89 (t, *J* = 9.56 Hz, 2H), 1.29–1.54 (m, 22H), 0.91 (t, *J* = 8.58 Hz, 3H); ^13^C-NMR (CD_3_OD) δ: 166.12, 164.41, 163.97, 133.43, 132.46, 131.29, 129.82, 129.60, 128.50, 128.06, 125.26, 122.37, 65.86, 56.11, 42.84, 35.31, 31.68, 29.37, 29.28, 29.24, 29.08, 28.96, 28.34, 25.78, 13.04; ESI-MS *m*/*z*: 509.36 [M + 1 − HCl]^+^. Anal. Calcd. for C_31_H_45_ClN_2_O_4_·0.2H_2_O: C 67.85%, H 8.34%, N 5.10%; found C 67.65%, H 8.41%, N 5.01%; MW: 545.15.

### 3.4. Materials and Cell Lines

RPMI medium 1640 and fetal bovine serum (FBS) were purchased from Gibco (Grand Island, NY, USA). 3-(4,5-Dimethylthiazol)-2,5-diphenyltetrazolium bromide (MTT) and propidium iodide (PI) were purchased from Sigma (St. Louis, MO, USA). Acridine orange (AO) and ethidium bromide (EB) were purchased from Amresco (Solon, OH, USA). The sources of primary antibodies used for western blotting: p21, cyclin B1, E-cadherin, β-actin and as well as the corresponding horseradish peroxidase-conjugated second antibodies were all obtained from Santa Cruz Biotechnologies (Santa Cruz, CA, USA). RIPA buffer, BCA assay kit, ECL plus reagents were purchased from Beyotime (Shanghai, China). SMMC-7721, HepG2 and H22 cells were purchased from the cells bank of the Chinese Academy of Science (Shanghai, China). Cells were maintained in RPMI medium 1640 supplemented with 10% FBS, 100 units/mL penicillin and 100 μg/mL streptomycin.

### 3.5. Cytotoxicity against Cancer Cell Lines

Antiproliferative ability of compound **3a** was evaluated in HepG2 and SMMC-7721 cells using MTT assay. In brief, cells were seeded into 96-well cell culture plates at a density of 5 × 10^3^ cells/well. After cells adherence, various concentrations of compound **3a** (0, 1, 5, 10, 30 and 50 μM) were added. After incubation for 48 h, 50 μL MTT (1 mg/mL) was added followed by incubation at 37 °C and 100 μL DMSO was added to solubilize the crystal products. The optical density (OD) was measured at a wavelength of 570 nm with a microplate reader (BioTek, Winooski, VT, USA). The experiments were repeated at least three times.

### 3.6. Cell Morphology Observation

The parameters of cells, morphology and size, reflect the physiological and functional state of cells. Hence, observing cells morphological changes is the easiest and most intuitive way to analyze the state of cells. Cells were seeded into 24-well plates at a density of 4 × 10^3^ cells/well. Various concentrations of compound **3a** were added for 48 h incubation. Then, cell morphological changes were observed using an inverted biological microscope (20×).

### 3.7. Cellular Apoptotic Evaluation

HepG2 cells were seeded in 96 well plates (6 × 10^3^ cells/well), cultured for 24 h to obtain a confluent monolayer and then treated with various concentrations of tested compounds for 48 h. Cells were incubated with acridine orange (50 μM)/ethidium bromide (50 μM) for 30 min, then washed with PBS to remove unbound dyes. Images were obtained on the High Content Screening (HCS, ArrayScan, ThermoFisher, Pittsburgh, PA, USA) reader using Target Activation BioApplication software (ThermoFisher, Pittsburgh, PA, USA).

### 3.8. Cell Cycle Analysis

Cell cycle distribution was measured by flow cytometry with propidium iodide (PI) staining. Cells were seeded into 6-wells plates, followed by the addition of various concentrations of compound **3a**. After 48 h, cells were collected and fixed in ice-cold 70% ethanol. After washed twice with ice-cold phosphate buffered saline (PBS), cells were treated with 50 μg/mL RNase A and stained with 25 μg/mL PI. Cell cycle distributions were analyzed by flow cytometry (BD FACSVerse, San Jose, CA, USA). Apoptotic cells show an appearance of a sub-G1 (<2N ploidy) peak.

### 3.9. Migration Assay In Vitro

The wound scratch assay is considered as a convenient and inexpensive method for analysis of cell migration in vitro [42]. The cells were plated into 24-well plates at a density of 2.0 × 10^5^ cells/well and grown to create a confluent monolayer. Then, cells were scratched in a straight line using a 10 μL micropipette tip and washed with PBS to remove floating cells. After they were photographed, various concentrations of compound **3a** were added to serum-free medium to treat cells for 24 h and photographed.

### 3.10. Transwell Invasion Assay

The invasion assay was performed using 24-well transwell inserts containing an 8 μM pore polycarbonate membrane (Corning, Corning, NY, USA) coated with matrigel (BD Biosciences, San Jose, CA, USA) [43]. Briefly, 4 × 10^4^ cells suspended was added to the upper compartment of the transwell inserts. Various concentrations of compound **3a** were added to cells to incubate at 37 °C for 24 h. The non-invaded cells were removed and the invaded cells were stained with 0.1% crystal violet. Cells were photographed, and glacial acetic acid was added to 24-well transwell inserts to release the bound dye. The optical density (OD) was measured at 570 nm with a microplate reader (BioTek, Winooski, VT, USA). The experiments were repeated at least three times.

### 3.11. Western Blotting

Cells were collected and washed with ice-cold PBS. The prepared cells were lysed with RIPA buffer (Beyotime) at 4 °C for 1 h and centrifuged at 12,000 × *g* for 10 min at 4 °C. The total protein concentration was determined by BCA assay kit, and equivalent total proteins were mixed with 5× loading buffer and boiled at 100 °C for 5 min. The samples were separated by 12% SDS-PAGE, and transferred onto 0.45 μm PVDF membranes. After blockage, membranes were incubated with corresponding primary antibodies. Appropriate HRP conjugated secondary antibody was used. Protein bands were detected by using the BeyoECL plus reagents (Beyotime).

### 3.12. Evaluation of Antitumor Effects In Vivo

Swiss mice (6 to 8 weeks old) were purchased from the Laboratory Animal Center of Henan (Zhengzhou, China). All animal procedures were performed following the protocol approved by the Institutional Animal Care and Use Committee at Henan University (approval ID: HUSOM-2017-165; date of approval: 2017/1/9). 2.5 × 10^6^ H22 cells were injected subcutaneously in the right flank of the Swiss mice for tumor development [44]. After injection for seven days, tumor-bearing mice were randomly divided into the following three groups (*n* = 10 mice per group): a negative control group, compound **3a** group and amonafide group. Then, tumor-bearing mice were treated respectively by normal saline (control group), compound **3a** (5 mg/kg) and amonafide (5 mg/kg) once every day for seven consecutive days via tail vein. Acute toxicity was used to determine the suitable dose, and mice were injected by compound **3a** via tail vein once every day for seven consecutive days. Tumors were isolated from mice, weighed, fixed in formalin, and section slices were stained with hematoxylin and eosin (H&E) to detect the growth of tumor cells. The tumor-inhibition rate was calculated as follows: tumor-inhibition rate = [(weight_control_ − weight_drug_)/weight_control_] × 100%.

For tumor metastasis, 2.5 × 10^6^ H22 cells were injected through the tail vein of Swiss mice. The grouping and dosing were the same as before. Mice were injected by tail vein daily for seven days as described previously. Mice were allowed to diet freely, and weighted at a fixed time daily. Lungs were isolated from mice, weighed, fixed in formalin, and section slices were stained with H&E. Lung metastatic nodules were counted and inhibition rate of lung metastasis was calculated as follows: inhibition rate = [(lung nodules number_control_ − lung nodules number_drug_)/lung nodules number_control_] × 100%.

### 3.13. Systemic Toxicity and Histopathological Evaluation

Heart, liver, spleen, lung and kidney of treated-mice were collected and weighted. The visceral indexes and histopathology were investigated for systemic toxicity evaluation. Visceral index (%) = (viscera weight/body weight) × 100%. Collected tumor from each group for histopathological evaluation at tested times and put them in 10% formaldehyde to fix. Tumor sections were stained with H&E for examination of any histopathological changes.

### 3.14. Data Analysis

All data are presented as the mean ± SD, and analyzed using Student’s *t*-test or analysis of variance (ANOVA) followed by *q*-test: compared with control, * *p* < 0.05, ** *p* < 0.01, *** *p* < 0.001 as significant.

## 4. Conclusions

In summary, this study reported the synthesis of two subsets of naphthalimide derivatives. Compounds **3a**–**e** with a fused thiazole ring generally showed better in vitro antitumor activity than the corresponding naphthalimide derivatives with a formic acid ester at the 4-position. Compound **3a** exerted potent effects against two HCC models of primary tumor and lung metastasis. Cell death profile research revealed that compound **3a** inhibited cancerous liver cell growth mainly by G2/M phase arrest, accompanied by the up-regulated protein expression of cyclin B1, CDK1 and p21. Meanwhile, **3a** inhibited cell migration by elevating the expression of E-cadherin and attenuating the expression of integrin α6. Importantly, **3a** had no obvious systemic toxicity at the therapeutic dose, indicating it was worthwhile for further development.

## Figures and Tables

**Table 1:** Inhibitory results of target compounds against four cancer cell lines.

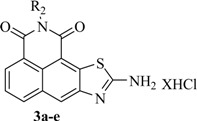		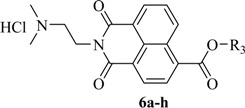			
Compound	R_2_ (R_3_), X=	IC_50_ (µM) ^a^			
		SMMC-7721	HepG2	HCT-116	K562
**Amonafide**		10.32 ± 1.04	11.67 ± 1.45	6.86 ± 0.79	10.10 ± 1.67
**3a**	-(CH_2_)_3_NH_2_, X = 3	6.03 ± 0.84	9.33 ± 0.95	11.71 ± 1.06	27.50 ± 2.31
**3b**	-(CH_2_)_4_NH_2_, X = 3	21.84 ± 1.85	16.48 ± 1.21	30.42 ± 2.49	42.50 ± 3.87
**3c**	-(CH_2_)_3_NH(CH_2_)_3_NH_2_, X = 4	1.61 ± 0.13	4.67 ± 0.31	3.81 ± 0.25	12.41 ± 1.56
**3d**	-(CH_2_)_4_NH(CH_2_)_4_NH_2_, X = 4	26.86 ± 1.73	>50	15.79 ± 0.98	>50
**3e**	-(CH_2_)_4_NH(CH_2_)_4_NH, X = 4	10.07 ± 0.89	19.18 ± 1.84	3.18 ± 0.27	27.18 ± 2.39
**6a**	-CH_3_, X = 1	22.06 ± 1.77	22.11 ± 1.69	46.48 ± 3.93	>50
**6b**	-CH_2_CH_3_, X = 1	13.66 ± 1.04	25.85 ± 2.48	46.76 ± 3.56	42.99 ± 2.54
**6c**	-*n*-C_3_H_7_, X = 1	>50	>50	>50	>50
**6d**	-*n*-C_4_H_9_, X = 1	>50	>50	>50	>50
**6e**	-*n*-C_5_H_11_, X = 1	>50	>50	>50	>50
**6f**	-*n*-C_6_H_13_, X = 1	>50	>50	>50	>50
**6g**	-*n*-C_8_H_17_, X = 1	>50	>50	>50	>50
**6h**	-*n*-C_14_H_29_, X = 1	>50	>50	>50	>50

^a^ IC_50_ values represent the concentration causing 50% growth inhibition. They were determined by the linear regression method. Each sample is the mean of three independent experiments.
